# Additive Manufacturing and Characterization of Metal Particulate Reinforced Polylactic Acid (PLA) Polymer Composites

**DOI:** 10.3390/polym13203545

**Published:** 2021-10-14

**Authors:** Ved S. Vakharia, Lily Kuentz, Anton Salem, Michael C. Halbig, Jonathan A. Salem, Mrityunjay Singh

**Affiliations:** 1 NASA Pathway Intern, Department of Mechanical and Aerospace Engineering, University of California, San Diego, CA 92092, USA; ved.vakharia@gmail.com; 2 NASA Intern Currently at Department of Geology, University of Oregon, Eugene, OR 97403, USA; lilykuentz@gmail.com; 3 NASA Intern Currently at VulcanForms, Inc., Burlington, MA 01803, USA; antonsalem1@gmail.com; 4 NASA Glenn Research Center, Cleveland, OH 44135, USA; jonathan.a.salem@nasa.gov; 5 Ohio Aerospace Institute, Cleveland, OH 44142, USA

**Keywords:** Polylactic Acid (PLA), 3-D printing, polymer composites, multifunctionality, fused filament fabrication, metal-reinforced PLA, mechanical properties

## Abstract

Affordable commercial desktop 3-D printers and filaments have introduced additive manufacturing to all disciplines of science and engineering. With rapid innovations in 3-D printing technology and new filament materials, material vendors are offering specialty multifunctional metal-reinforced polymers with unique properties. Studies are necessary to understand the effects of filament composition, metal reinforcements, and print parameters on microstructure and mechanical behavior. In this study, densities, metal vol%, metal cross-sectional area %, and microstructure of various metal-reinforced Polylactic Acid (PLA) filaments were characterized by multiple methods. Comparisons are made between polymer microstructures before and after printing, and the effect of printing on the metal-polymer interface adhesion has been demonstrated. Tensile response and fracture toughness as a function of metal vol% and print height was determined. Tensile and fracture toughness tests show that PLA filaments containing approximately 36 vol% of bronze or copper particles significantly reduce mechanical properties. The mechanical response of PLA with 12 and 18 vol% of magnetic iron and stainless steel particles, respectively, is similar to that of pure PLA with a slight decrease in ultimate tensile strength and fracture toughness. These results show the potential for tailoring the concentration of metal reinforcements to provide multi-functionality without sacrificing mechanical properties.

## 1. Introduction

3-D printing is a fast-evolving technology due to its ease of access and usefulness in various avenues of science and engineering. In contrast to traditional subtractive manufacturing techniques, additive manufacturing creates objects in an additive process through a layer-by-layer joining method. This method presents the benefit of materials savings, as well as new design approaches. The introduction of 3-D printing has pushed industries, such as automotive, aerospace, energy, and medical, to new heights [1,2]. Recent technological advancements center on the implementation of multifunctional materials [3,4,5]. With the development of new lightweight multifunctional components and systems, there is a need for new 3-D printable materials and robust manufacturing processes.

In just a few years, there have been significant advances in the development of polymer 3-D printing systems ranging from commercially available high-end fused deposition modeling (FDM), also referred to as fused filament fabrication (FFF) machines to desktop 3-D printers. 3-D printing can be advantageous to traditional manufacturing processes depending on manufacturing constraints. 3-D printing technologies allow for far more complex geometries, eliminate the need for tooling, and their versatility introduces printing with varying materials and parameters. However, many low-cost printers can only fabricate materials from acrylonitrile butadiene styrene (ABS) and polylactic acid (PLA) systems. As a result, these are the most studied 3-D printed materials [6,7,8]; however, low-cost manufacturing could benefit from multifunctional ABS and PLA material production capability.

There is literature available on the PLA material system for 3-D printing. Tymrak et al. [9] measured the tensile strength and elastic modulus of printed components made from low-cost 3-D printers. They found an average tensile strength and elastic modulus for PLA to be 56.6 MPa and 3368 MPa, respectively. A life cycle analysis performed by Wittbrodt et al. and Kreiger et al. [10,11] suggests that 3-D printing as a manufacturing method has a lower environmental impact than that of conventional, subtractive manufacturing for a variety of products. Additive manufacturing can reuse reclaimed materials as a filament in a variety of manufacturing methods [12]. However, there is an informational void for modified PLA systems, particularly those with metallic particle reinforcements. Other reinforcement materials such as synthetic or natural fibers, ceramics, and metals have been explored [13]. Namiki et al. impregnated carbon fiber into PLA filament to take advantage of continuous fiber reinforcement [14]. Several other studies have similarly investigated carbon fiber, and modified carbon fiber reinforced PLA filaments [15,16,17,18,19,20,21,22]. A previous study characterized metal-reinforced PLA filaments, but Fafenrot et al. only explored bronze and magnetic iron reinforced PLA [23]. A very recent study successfully implemented continuous metal fibers (copper and steel wire) into PLA filaments and enhanced the composite’s mechanical properties [24]. Liu et al. characterized the mechanical response of metal-based PLA filaments; specifically, copper and aluminum powder. They found that enhanced strength is also possibly dependent on printing properties such as the raster angle [25].

Traditionally, PLA is one of the most commonly used polymers for 3-D printing. Recently, composite filament materials have emerged, such as particulate reinforced PLA. The additions to the PLA aim to improve the multi-functionality by affecting such properties as strength, thermal conductivity, electrical conductivity, magnetic attraction, and radiation shielding. Metal-oxide semiconductors have been manufactured from sintered 3-D printed materials [26], and filaments with TiO_2_ and MoO_3_ were explored for their antimicrobial and antifungal properties [27]. Multiple groups pursued multifunctionality to try and introduce electroconductive properties to 3-D printed materials [28,29,30]. However, the specifics of these materials are highly variable. Cost can range from expensive industrial grade to inexpensive pellets (approximately $1/lb) that can be used to create filament. Density, and perhaps most significantly, metal volume percent, can vary from manufacturer to manufacturer. These material details play a vital role in determining the mechanical, thermal, and electrical properties of PLA-based systems and, therefore, govern a product’s design.

In this study, PLA was printed using desktop 3-D printers. The microstructure, density, tensile strength, elastic modulus, and fracture toughness of these materials were determined as a function of their print height and metallic composition. This research aims to determine the role that starting filaments and print height play in determining the mechanical properties of 3-D printed materials. Through the thorough characterization and better understanding of the commercially available filaments and the resultant printed material properties, these composite materials can be better tailored to improve specific properties to be used in multifunctional applications.

## 2. Materials and Methods

**Figure 1:**
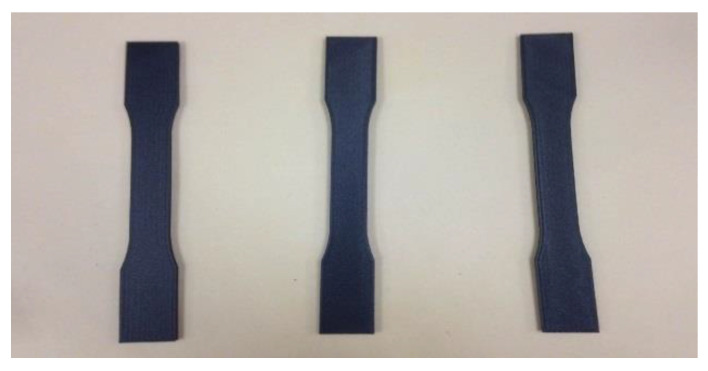
ASTM D 638 Tensile Specimens (6 inches long).

**Figure 2:**
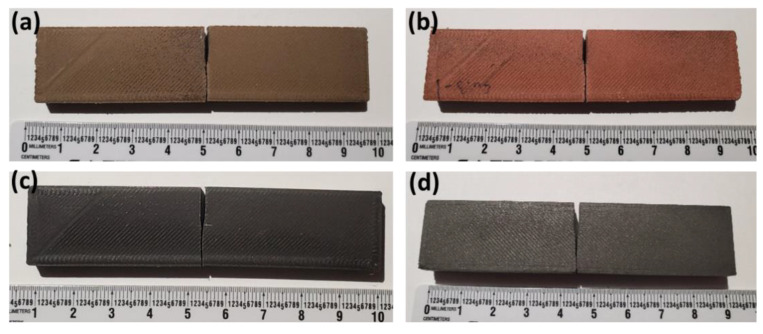
(**a**) Br-PLA, (**b**) Cu-PLA, (**c**), MI-PLA, (**d**) and SS-PLA specimens after undergoing fracture toughness testing.

This study used commercially available Makerbot Replicator 2X (TYPE, MakerBot, Brooklyn, New York, NY, USA) and Orion Delta 3-D printers (TYPE, SeeMeCNC, Ligonier, IND, USA). These machines were used to manufacture ASTM D 638 [31] tensile coupons (Figure 1), as well as fracture toughness specimens (Figure 2). These specimens were printed with 0.1 mm, 0.2 mm, 0.3 mm, and 0.4 mm layer heights, with an overall thickness between 9.6 and 11.2 mm. In addition to varying the layer height of the printed specimens, the print material was changed. Typical extruder temperatures were 200–220 °C and bed temperatures were 45–60 °C depending on the filaments. Different materials used in this study were pure PLA (Makerbot), bronze-reinforced PLA (colorFabb), copper-reinforced PLA (colorFabb), magnetic iron-reinforced PLA (Proto-pasta), and stainless steel-reinforced PLA (Proto-pasta). These materials will hereby be referred to as PLA, Br-PLA, Cu-PLA, MI-PLA, and SS-PLA, respectively. Three tensile coupons and three fracture toughness specimens of each layer height and material type were printed and tested.

Prior to testing, the bulk density of each material was measured along with that from the Archimedes buoyancy method. Through thermogravimetric analysis (TGA) of bulk filament pieces, metallic particle weight percentage was obtained, from which metallic volume percentage was calculated. Samples from printed coupons and as-supplied filament were mounted in epoxy and polished to allow for microstructural analysis of the cross-sections using an optical microscope. Measurements of metallic particle area percentage and porosity with the PLA were conducted on the optical micrographs using ImageJ (National Institutes of Health, Bethesda, MD, USA)).

For tensile testing, the strains were determined by using photogrammetry. The elastic moduli of materials were determined in two different manners: dynamically and mechanically. The dynamic testing consisted of impulse excitation in nominal conformance with ASTM C1259 [32]. Mechanical modulus was determined from the tensile data. Generally, dynamic and mechanical results were similar, and the dynamic testing ceased. Fracture toughness was determined according to D5045 [33]. The thickness, width, and crack length of each specimen were approximately 10, 20, and 9 mm, respectively. Some of the thickness or plasticity criteria were not fulfilled; however, additional specimens of some materials tested with a greater thickness (10 mm) resulted in similar values, implying reasonable trends in the data and conclusions.

## 3. Results and Discussions

### 3.1. Material Characterization

**Figure 3:**
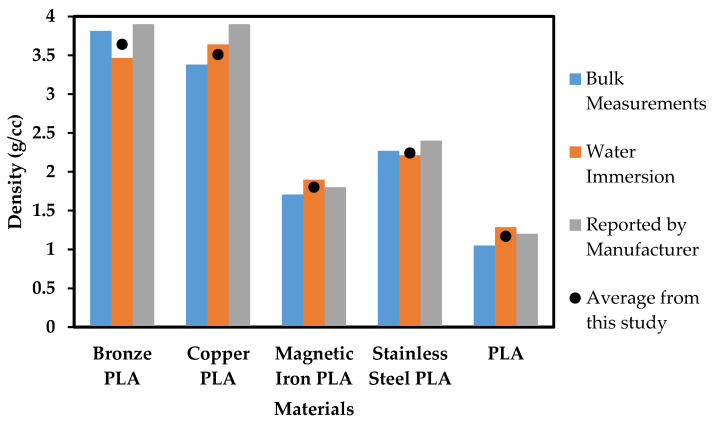
Filament densities obtained from multiple methods and reported by filament manufacturers.

Figure 3 compares density measurements for all materials used in this study. There is a discrepancy between “in-house” water immersion density measurements and those provided by the filament producer, particularly the 0.26 g/cc and 0.43 g/cc difference present in the Br-PLA and Cu-PLA materials, respectively. The difference between filament producer-provided measurements and those carried out in this study is at least 0.1 g/cc for every material. It is critical to have an accurate density of source materials before designing a 3-D printed structure, and therefore the values need to be assessed and confirmed. This information can be found by simply characterizing material before use and then designing structures accordingly.

**Figure 4:**
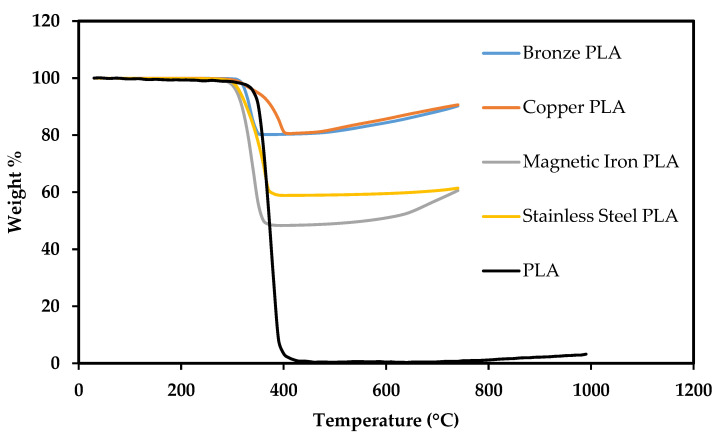
TGA results of materials conducted in air.

Figure 4 shows the TGA results, performed in air, of the materials used in this study. By approximately 400 °C, the PLA has burned away, and the remaining weight can be associated with the metallic particle reinforcements. For example, approximately 80 wt% of bronze and copper are remaining once the PLA is removed from each sample at 400 °C. The metal wt% and associated vol% are displayed in Table 1.

Fafenrot et al. conducted a similar study on Br-PLA and MI-PLA filaments that were obtained from the same sources as in this study. They observed very similar metal vol% of 36 and 12, respectively. Their calculations were also conducted via TGA measurements [23].

Br-PLA and Cu-PLA exhibit a much higher metal vol% than MI-PLA or SS-PLA. Direct comparisons of mechanical strength between these materials cannot be conducted from these measurements, in part due to density difference and varying reinforcement vol% values. It can be rationally predicted that material with 36 vol% copper is far less likely to behave as pure PLA than a material with only 11% magnetic iron. The volume of metal reinforcement should significantly affect mechanical properties in comparison to pure PLA. The multifunctionality of material is significantly dependent on its composition and, in this case, the amount of metal reinforcement in PLA. An objective of this research is to determine if multifunctionality may be tailored to a specific purpose if this metal vol% can be controlled.

**Figure 5:**
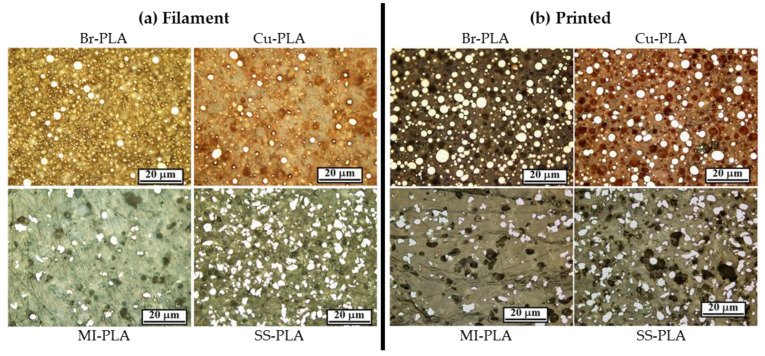
Micrographs of Br-PLA, Cu-PLA, MI-PLA, and SS-PLA (**a**) filaments before printing, and (**b**) parts after printing.

**Figure 6:**
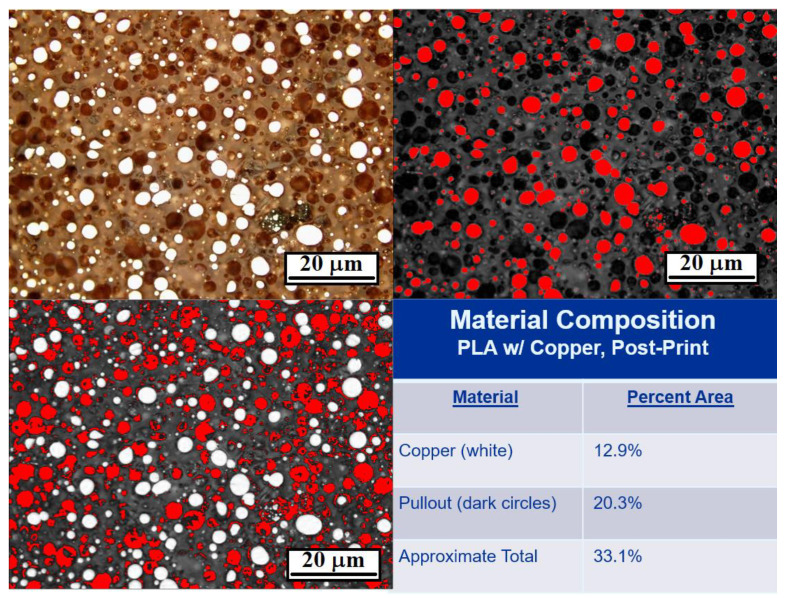
Area% calculation of Cu- PLA (post-print) where bright white spots (metal particles) are added with dark spots (pullout locations) to obtain total area% particles (performed with ImageJ).

**Figure 7:**
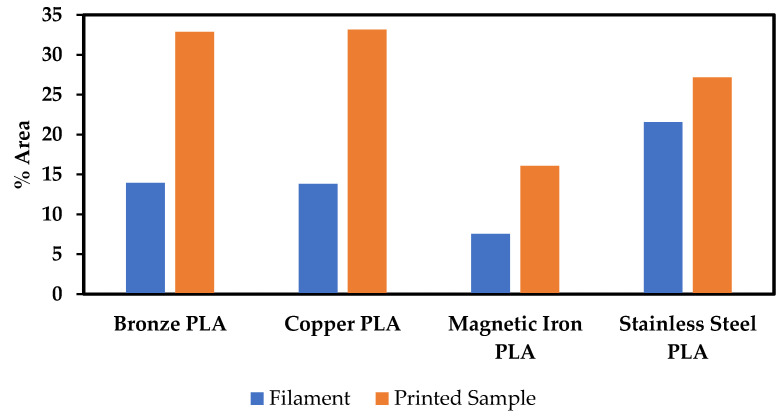
The measured area percent of Br-PLA, Cu-PLA, MI-PLA, and SS-PLA before and after printing.

Figure 5a shows micrographs for Br-PLA, Cu-PLA, MI-PLA, and SS-PLA filament (before printing), and Figure 5b shows micrographs of the same materials after printing. The bright, white regions in the micrographs are the metallic particles, with the surrounding material being PLA. In all the micrographs in Figure 5a,b, except perhaps the Br-PLA filament prior to printing (Figure 5a), there are dark spots that have shapes resembling those of the metallic particles. Due to the size and shape similarities between them, we hypothesize these dark spots are created from metallic particles pulling out of the PLA rather than the porosity created during printing. This most likely occurred during the polishing of the mounted samples. The abrasive surface of the polishing pads caught onto the edges of the hard metal particles sticking out above the soft PLA, and pulled them out. Therefore, area percentages of metallic particles were obtained by combining the approximate amounts of bright and dark spots as shown in Figure 6. The area percentages determined from the micrographs from Figure 5a,b can be seen in Figure 7 and Table 2.

The cross-sectional area % measured (using ImageJ) for particles in the printed materials in Figure 5b is similar to the vol% of metallic particles in these filaments as obtained through TGA experiments; however, that is not the case for results obtained from analyses of the filament micrographs displayed in Figure 5a. Since TGA experiments were conducted on the as-received filament, it should be expected that the metallic particle area % of the filament micrographs (when including the pullout amounts) should be similar to the vol% obtained from TGA measurements.

The possibility that gravity effects during the printing of the bulk samples could cause the discrepancies between the metal area % determined from the micrographs and the vol% calculated from the TGA experiments was considered. These samples were created with commercially available desktop 3-D printers that were not designed to print polymer filament reinforced with metallic particles. As the filament is heated at the printer head, the decreased viscosity may allow for metal particles just above the heated region to migrate down to the print head and be dispersed into the more viscous regions of the filament. In other words, the structure maintaining the position of these metal particles loses its strength, allowing for the metal to migrate in the direction forced by gravity. However, the gravity effect is most likely occurring within a printed layer height, which should only affect the location and not the amount of detectable reinforcement. All printed layers are within the polished plane in the micrograph, and therefore the metallic particles should be visible. There are also certain issues in changing the nozzle diameter due to abrasion as well as sticking of some particulates in the nozzle orifice. These factors should be evaluated carefully in future investigations.

In addition, the printing procedure may cause the plastic filament to shrink, causing the metallic particles to occupy a higher area % than they did before printing. However, PLA has proven to have minor warping or shrinking issues upon cooling, unlike ABS, which warps drastically after printing [34,35].

There seems to be significantly more metal reinforcement in printed samples. The most likely explanation of why filament cross-sections show less area % than printed parts is that the printing process has a beneficial effect on the adhesion between the metallic reinforcement and the PLA. The mounting and polishing procedure to prepare polished sections was consistent between filaments and printed samples, yet less reinforcement is detected in the filament. The PLA was exposed to a different temperature and cooling time in each print, possibly affecting the PLA interface around the particles.

**Figure 8:**
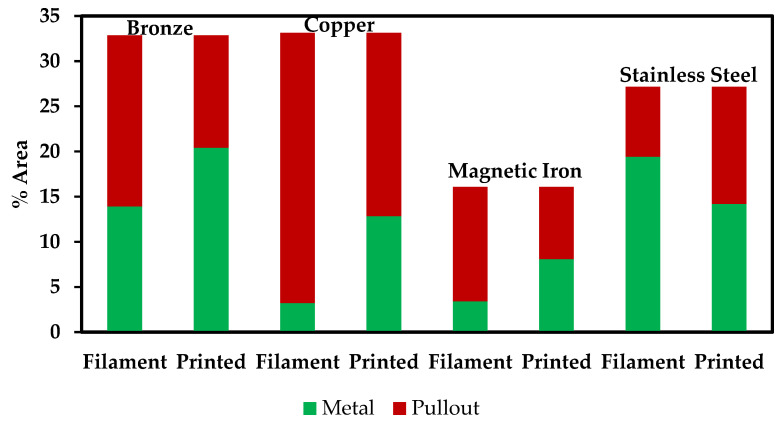
A visualization of the data presented in Table 3 showing the area percentage of total reinforcement observed in filament and printed micrographs.

Gupta et al. performed tribological studies on printed samples of the same materials used in this study and found that the presence of metallic reinforcements, regardless of the metal, decreases the wear rate and coefficient observed in PLA [36]. Studies weren’t conducted to compare the tribological behavior between the filament and the printed sample of a given material. However, with the increase in pullout observed in this study, we expect the wear rate to be higher in filaments than in printed samples. The micrographs in Figure 5a are blurrier than those in Figure 5b due to the polishing finish, and this limits the ability to recognize all of the pullout locations of the metallic particles. That could also be an effect of fewer particles being present, resulting in a smoothing of the matrix. Therefore, a different method was used to estimate the amount of pullout in the polished filament samples. If it is assumed that the reinforcement amount is correctly identified in the printed samples (taking into account observed pullout) and through the TGA experiments, then we can calculate how much reinforcement was pulled out of the filaments during the polishing procedure. That data is shown in Table 3 and Figure 8. Except for SS-PLA, there was less pullout detected in the printed samples. To obtain the best accuracy in future assessments of particulate vol%, multiple images of each sample will be assessed in order to provide a larger database. In addition, performing TGA on the printed material should provide optimal information about the metallic particle content.

This pullout observation supports the hypothesis that PLA-metal interface adhesion may be weak, especially before undergoing a heating and cooling procedure via printing, and should be optimized. It is disconcerting that a simple polishing procedure easily removes at least half of the metallic particles from printed samples. The weak PLA-metal interface adhesion does not provide promise for the use of these materials on exposed surfaces that may experience friction with their surroundings, hindering the design process for these materials. As the filament is heated and printed, it may be unavoidable for the metallic particles to move around in the PLA. However, the adhesion at the PLA-metal interface can be improved. Optimization of print temperature, extrusion rate, raster angle, and bed temperature may improve interface adhesion.

### 3.2. Mechanical Properties

**Figure 9:**
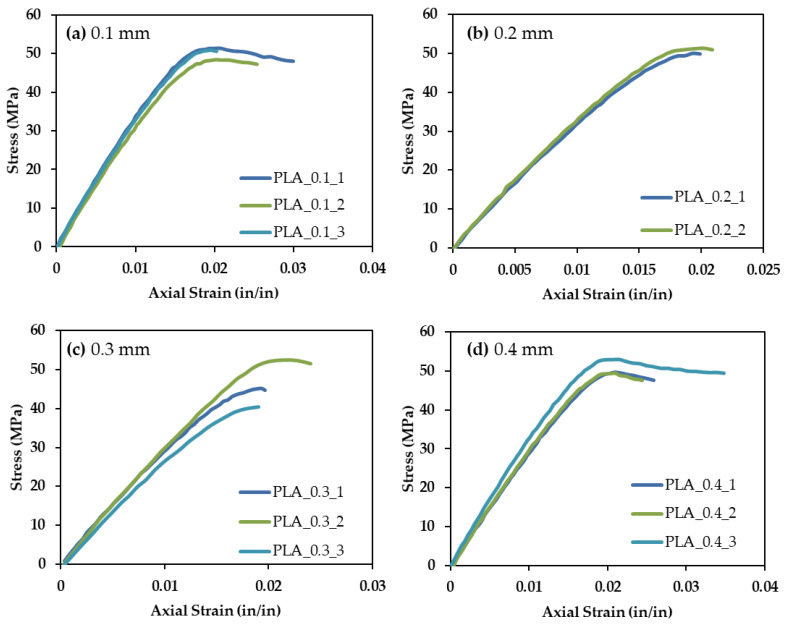
Tensile data for PLA with a print layer height of (**a**) 0.1 mm, (**b**) 0.2 mm, (**c**) 0.3 mm, and (**d**) 0.4 mm.

**Figure 10:**
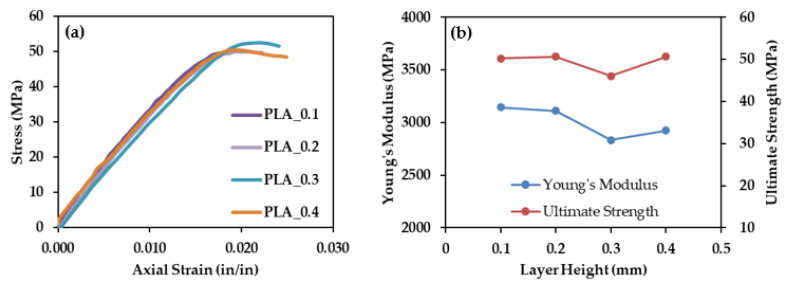
(**a**) Average tensile response and (**b**) Young’s modulus and Ultimate Strength for PLA printed at 0.1, 0.2, 0.3, and 0.4 mm print layer heights.

Figure 9a–d show the data obtained from tensile testing for PLA samples printed with 0.1, 0.2, 0.3, and 0.4 mm layers, respectively. Three samples of each material were tested. One test of PLA printed with a print layer height of 0.2 mm failed prematurely due to a poor experimental setup. The overall results are consistent and provide confidence in the repeatability of the printing process. Strain to failure is variable; however, this is common for polymeric materials. Figure 10a shows the average tensile response of each of the four PLA print heights. The Young’s modulus of each material was calculated from the slope of the linear, elastic portion of the tensile response. The highest stress withstood by the material is taken as the ultimate strength. Figure 10b shows Young’s moduli and ultimate tensile strengths of the same samples. A slight decrease in modulus with print layer height is exhibited; however, little effect is seen on ultimate strength. PLA behaves consistently, regardless of print height. Therefore, metal-reinforced PLA can be compared to PLA printed at any of these four print heights. This consistency, regardless of print height, is not the case for all standard 3-D printed materials. For example, a previous study showed that ABS layer height plays a critical role in determining the 3-D printed polymer’s tensile performance [37]. Once again, material choice proves to be a governing parameter when designing 3-D printed structures.

**Figure 11:**
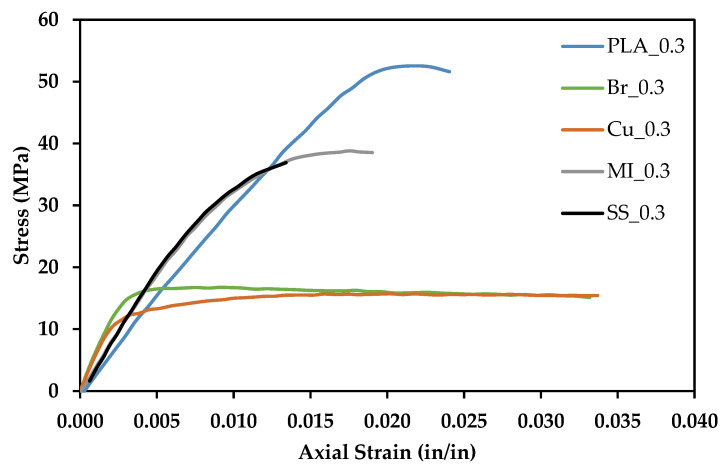
Average tensile response for PLA and metal reinforced PLA at 0.3 mm print layer height.

Figure 11 shows the average tensile response for metal-reinforced PLA samples printed at 0.3 mm print height compared to PLA samples printed at 0.3 mm print height. All the materials exhibit significant deformation prior to failure, with the Br-PLA and Cu-PLA exhibiting the largest strains (>3%). All metal-filled materials are weaker than pure PLA.

**Figure 12:**
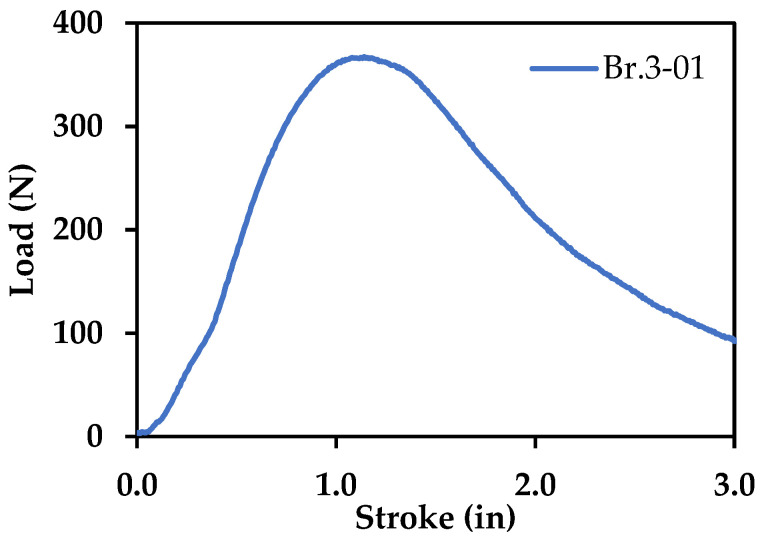
Data obtained from a three-point flexural test on Br-PLA.

**Figure 13:**
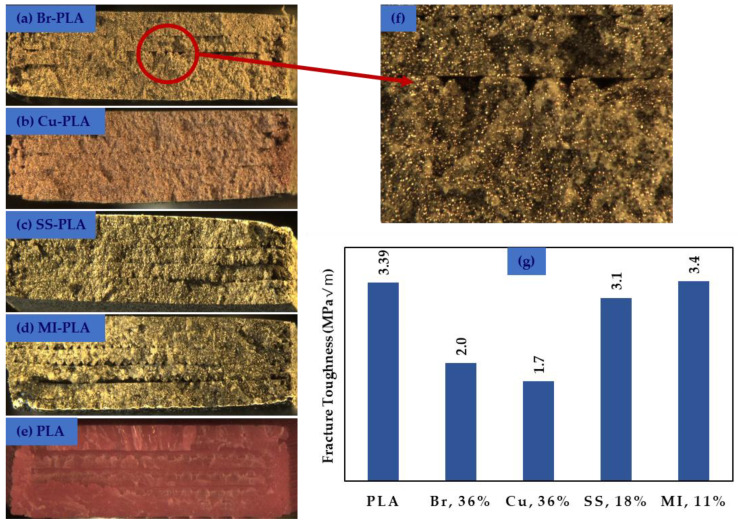
Fracture surfaces of (**a**) Br-PLA, (**b**) Cu-PLA, (**c**) SS-PLA, (**d**) MI-PLA, and (**e**) pure PLA, printed with a 0.3 mm print layer height. Br-PLA is shown at higher magnification in (**f**). (**g**) Corresponding fracture toughness values for materials (printed at a 0.3 mm layer height).

Fracture toughness was calculated with Equation (1),

(1) $$K_{Q}=\left(\frac{6P_{Q}\sqrt{a}}{BW}\right)\frac{1.99-\frac{a}{W}\left(1-\frac{a}{W}\right)\left(2.15-3.93\frac{a}{W}+2.7{\left(\frac{a}{W}\right)}^{2}\right)}{\left(1+2\frac{a}{W}\right){\left(1-\frac{a}{W}\right)}^{\frac{3}{2}}}\;,$$

where *P_Q_* is the applied load, *B* is the thickness of the specimen, *a* is the crack length, and *W* is the width of the specimen. An example of the data obtained from the three-point flexural tests used to calculate fracture toughness is shown in Figure 12. Figure 13a–e show fracture surfaces for all materials printed with a 0.3 mm print layer height, with Figure 13f showing the fracture surface of Br-PLA at a higher magnification. Print layers in each material are clearly visible, but only pure PLA is free of porosity between layers. Figure 13g displays the average fracture toughness of the materials investigated in this study. All images and data shown in Figure 13 are obtained from prints utilizing a 0.3 mm print layer height.

In all mechanical tests, SS-PLA and MI-PLA performed nearly as well as pure PLA. These three material compositions show similar tensile responses, but the presence of metallic particles causes a lower ultimate strength and earlier onset of plastic deformation. In contrast, there is a significant and noticeable change in mechanical behavior in Br-PLA and Cu-PLA. These two material compositions have much lower ultimate strength and fracture toughness values. Large additions of metal particles in the Br- and Cu-PLA may have resulted in lower fracture toughness due to the pull-out of the particles from the PLA.

The Br-PLA and Cu-PLA filaments contained approximately 36 metal vol%, while the MI-PLA and SS-PLA only contained 11 and 18 metal vol%, respectively. The high vol% addition of bronze and copper plays a vital role in causing this change in mechanical response. The significant presence of bronze and copper transforms PLA into a significantly more ductile structural material at a high cost of reduced ultimate strength and fracture toughness. They begin to deform plastically immediately after a strain is applied. These two materials show potential for novel 3-D printed structural design if the goal is for a more resilient (yet weaker) material.

However, a more beneficial scenario would be if the presence of metal additions only introduced new optical, magnetic, electrical, or thermal properties without affecting the mechanical properties of the polymer. Interestingly, the presence of magnetic iron and stainless steel do not have drastic effects on the tensile performance of PLA. There is a slight increase in the Young’s modulus of MI-PLA and SS-PLA. However, since the ultimate strength decreases with metal addition, the increased stiffness is not necessarily a positive change in tensile response. The presence of magnetic iron or stainless steel does not drastically affect the tensile response of PLA due to its small concentration. Behavior that is more similar to pure PLA is beneficial because it allows the introduction of multifunctionality without sacrificing mechanical strength or toughness. Perhaps a lower vol% of bronze or copper in the PLA would result in a similar tensile response to MI-PLA and SS-PLA filaments.

Table 4 shows the Young’s modulus, ultimate strength, Poisson’s ratio, and fracture toughness for each material. In every case, metallic particle reinforcement increases the Young’s modulus, leading to a slightly stiffer material and a decrease in ultimate strength and fracture toughness. Fafenrot et al. observed similar tensile responses in their Br-PLA and MI-PLA filaments. Their data shows Br-PLA deforming plastically almost immediately after the strain is applied, while MI-PLA behaves similar to pure PLA but with a slightly earlier onset of plastic deformation. Both trends agree with those shown in this study. They also measured ultimate tensile strength for Br-PLA, MI-reinforced, and pure PLA processed using varying temperatures and nozzle diameters. Their data did vary with changing print parameters but did not stray much from an average value. Their values for ultimate tensile strength ranged between 12 and 20 MPa for Br-PLA, 35 and 40 MPa for MI-PLA, and 45 and 50 MPa for pure PLA filaments [22]. These values and trends are similar to those found in this study as well.

## 4. Conclusions

There is potential to use metal-reinforced PLA as a material when designing novel 3-D printed structures. Though they may exhibit a slightly lower tensile strength and fracture toughness than pure PLA, this difference can be considered an acceptable sacrifice for introducing multifunctionality to 3-D printed materials. This study shows that small amounts of metal addition in the form of particulates can introduce multifunctionality to a polymer filament without drastically changing its tensile properties.

In this study, comparisons between polymer microstructures before and after printing and the effect of printing on the metal-polymer interface adhesion have been demonstrated. A more significant number of metallic particles were observed in the printed PLA samples than in the pre-print filament. The disparity is possibly due to a weak metal-polymer interfacial adhesion allowing metal particles to be removed from the PLA substrate during polishing. Polishing can represent a situation in which the material surface experiences friction with its surroundings. It is detrimental for the printed materials to have their reinforcements so easily removed. The lack of metal to PLA adhesion introduces some variability to material properties, where designs are made assuming that most, if not all, metallic particles remain bonded to its polymer substrate. Perhaps this inconsistency can be avoided by using a more robust 3-D printer instead of the readily available desktop printers used in this study. However, a material with excellent metal-polymer interfacial adhesion would not present this problem. Further studies optimizing the print temperature, extrusion rate, and bed temperature can bring more insight into this metal-polymer interface.

PLA shows consistent tensile response, regardless of print layer height. Print heights of 0.1, 0.2, 0.3, and 0.4 mm layers all performed similarly in mechanical testing. Since there are unequal metal vol% amounts, a direct comparison between the metal-reinforced PLA materials could not be made. However, the large amount of bronze and copper additions caused the PLA to deform plastically almost immediately as the strain was introduced to the specimens. The high concentration of metallic particles also significantly lowered the fracture toughness of these samples. The low amounts of magnetic iron and stainless-steel addition to PLA prevented a drastic change from the mechanical response of pure PLA. Regardless of the metal, the presence of these additions caused the PLA to exhibit a lower ultimate strength and fracture toughness. As a result of a poor metal-polymer interface adhesion, the metallic particulates introduce significant porosity into the composite, which may be the prevailing reason for the poor mechanical performance. Further studies into composite printing parameters may reveal how to maximize the metal-polymer interface adhesion, minimize the porosity, and increase mechanical properties of pure PLA or better. In order to confirm the presence of significant multifunctionality, a wide variety of mechanical, thermal, magnetic, and electrical tests and other characterization need to be performed on these materials.

## Figures and Tables

**Table 1:** Metal wt% and vol% of materials obtained from TGA of as-received filament.

Material	Metal wt%	Metal vol%
Br-PLA	80.35%	36.02%
Cu-PLA	80.57%	36.41%
MI-PLA	48.33%	11.05%
SS-PLA	58.87%	18.09%
PLA	0.00%	0.00%

**Table 2:** The measured area percent of Bronze (Br), Copper (Cu), Magnetic Iron (MI), and Stainless Steel (SS) in reinforced PLA filament and printed samples.

Material	Metal Area%	Pullout Area%	Total Area%
Br-Filament	13.9%	N/A	13.9%
Br-Printed	20.4%	12.5%	32.9%
Cu-Filament	3.2%	10.6%	13.8%
Cu-Printed	12.9%	20.3%	33.2%
MI-Filament	3.4%	4.1%	7.5%
MI-Printed	8.1%	8.0%	16.1%
SS-Filament	19.4%	2.1%	21.5%
SS-Printed	14.2%	13.0%	27.2%

**Table 3:** Area % of total reinforcement observed in filament and printed micrographs. Pullout amounts in filament micrographs are estimated by subtracting the detected metallic particles from the total (metal + pullout) of their printed counterparts.

Material	Metal Area%	Pullout Area%	Total Area%	Metal Vol% (TGA)
Br-Filament	13.9%	19.0% *		36.02%
Br-Printed	20.4%	12.5%	32.9%	
Cu-Filament	3.2%	30.0% *		36.41%
Cu-Printed	12.9%	20.3%	33.2%	
MI-Filament	3.4%	12.7% *		11.05%
MI-Printed	8.1%	8.0%	16.1%	
SS-Filament	19.4%	7.8% *		18.09%
SS-Printed	14.2%	13.0%	27.2%	

* Calculated

**Table 4:** Average Young’s modulus (MPa), ultimate strength (MPa), Poisson’s ratio, and fracture toughness (MPa√m) for all materials used in this study.

Material	Young’s Modulus (MPa)	Ultimate Strength (MPa)	Poisson’s Ratio	Fracture Toughness (MPa√m)
Br-PLA (0.3 mm)	5401	17	0.29	2.0
Cu-PLA (0.3 mm)	4496	16	0.25	1.7
MI-PLA (0.3 mm)	3638	39	0.32	3.4
SS-PLA (0.3 mm)	3910	38	0.33	3.1
PLA (0.1 mm)	3145	50	0.33	N/A
PLA (0.2 mm)	3110	51	0.34	N/A
PLA (0.3 mm)	2835	46	0.33	3.39
PLA (0.4 mm)	2925	51	0.34	N/A

## Data Availability

Not applicable.
